# Spatiotemporal expression of *MYD88* gene in pigs from birth to adulthood

**DOI:** 10.1590/1678-4685-GMB-2017-0014

**Published:** 2018-01-22

**Authors:** LiNa Gan, WeiYun Qin, Sen Wu, ShengLong Wu, WenBin Bao

**Affiliations:** 1 Key Laboratory for Animal Genetics, Breeding, Reproduction and Molecular Design of Jiangsu Province, College of Animal Science and Technology, Yangzhou University, Yangzhou, China.; 2 Joint International Research Laboratory of Agriculture & Agri-Product Safety, Yangzhou University, Yangzhou, China.

**Keywords:** MYD88, pig, developmental expression, immune response

## Abstract

MYD88 plays an important role in the immune response against infections. To analyze *MYD88* gene expression during different stages of pig development, we used real-time PCR. *MYD88* was seen expressed in all tissues examined. *MYD88* expression in spleen, lungs, and thymus reached its highest value from 7 to 14 days of age and decreased thereafter. Expression in lymph nodes was high until 28 days of age and then it declined after weaning, with stable low levels in adult pigs. *MYD88* expression was high before 35 days of age in the small intestine (duodenum, jejunum, and ileum), where it reached its highest value from 7 to 14 days of age. *MYD88* expression in the small intestine declined post-weaning and remained relatively low during adulthood. The results of this study suggest that weaning stress and development of the immune system might be positively correlated with *MYD88* expression regulation. Moreover, this study provided evidence that the high expression of *MYD88* may diminish weaning stress and increase disease resistance in Meishan pigs.

## Introduction

Mammalian species have an innate and an acquired immune system. The innate immune system is the first line of defense against pathogens (Zhou *et al.*, 2015). The toll-like receptor (TLR) family is a group of pattern recognition receptors (PRRs) that play important roles in sensing pathogen-associated molecular patterns (PAMPs) in innate immunity (Wack and Gallorini, 2008). The TLR family consists of 13 members with different downstream effects. The myeloid differentiation factor 88 (MYD88) is an adaptor protein for TLR signaling, with the exception of TLR3 (Shchebliakov *et al.*, 2010; Serezani *et al.*, 2011). MYD88 is a soluble cytoplasmic protein that belongs to both the Toll/IL-1R (TIR) family and the death domain family. The protein contains three functional areas: an N-terminal DD, a middle region, and a C-terminal TIR domain (Naro and Sette, 2016). The MYD88 TIR domain combines the TLRs and the TIR domain of IL-1R, which activates Interleukin-1 Receptor-Associated-Kinase-1/4 (IRAK-1/4) and tumor necrosis factor receptor-associated factor-6 (TRAF-6), resulting in the activation of nuclear factor kappa B (NF-κB) and the release of pro-inflammatory factors and cellular mediators. The activation of the TLR/MyD88/NF-κB signal pathway leads to lymphocyte activation and increased synthesis of pro-inflammatory proteins (Janssens and Beyaert, 2003; Arancibia *et al.*, 2007; Gay *et al.*, 2014; Liu *et al.*, 2014; Su *et al.*, 2015), thereby leading to an acquired immune response.

Porcine *MYD88*, which is located on chromosome 13 and has a coding sequence region consisting of 882 nucleotides, encodes 293 amino acids with 87% to 88% homologies between porcine and human proteins. *MYD88* is widely expressed in several tissues, especially in immune and intestinal tissues (Tohno *et al.*, 2007; Li *et al.*, 2009). The expression pattern of the gene is related to the immune response triggered by bacterial infections. Porcine MYD88 is a key protein in the TLRs/IL-1R signaling pathway that sends inflammatory signals and enhances the intensity of the inflammatory response. Additionally, MYD88 triggers the release of intestinal inflammatory mediators (Sun *et al.*, 2015).

The signal transduction pathway mediated by MYD88 is involved in the occurrence and development of several diseases. At present, most studies focus on its immune regulation (Singh *et al.*, 2015) and pathological mechanisms (Parpaleix *et al.*, 2016). However, no study has assessed the expression pattern of *MYD88*. In this study, we used real-time PCR technology to quatify *MYD88* expression at eight post-natal developmental stages in twelve tissues of Meishan pigs. Our goal was to provide a theoretical basis for further research on the genetics of *MYD88* in pig breeding for disease resistance.

## Materials and Methods

### Ethics statement

The animal study proposal was approved by the Institutional Animal Care and Use Committee (IACUC) of the Yangzhou University Animal Experiments Ethics Committee (permit number: SYXK [Su] IACUC 2012-0029). All experimental procedures involving piglets were performed in accordance with the Regulations for the Administration of Affairs Concerning Experimental Animals and approved by the State Council of People's Republic of China.

### Materials

Meishan pigs obtained from the Meishan Pigs Conservation Breeding Company (Jiangsu China) were used in this study. All the experimental pigs were maintained under standard piggery conditions. The animals had *ad libitum* access to a commercial-type compound feed with 21.7% crude protein and no antimicrobial additives or organic acids. We selected one animal from five litters at different developmental stages (newborn, 7-day old, 14-day old, 21-day old, 28-day old, 35-day old, 3-month old, and 6-month old). A total of 40 animals (five animals per group) were used, and the animals at the same developmental stage had similar characteristics (e.g., size and weight). Animals were electrically stunned (300 V for 5 s) and bled by heart puncture under the left armpit. Tissue samples from heart, liver, spleen, lung, kidney, stomach, muscle, thymus, lymph, duodenum, jejunum, and ileum were collected, frozen in liquid nitrogen and stored at -80 °C. All the experiments were conducted in the Yangzhou Key Laboratory of Animal Genetics and Breeding of Yangzhou University (Jiangsu, China).

### RNA extraction and reverse transcription

Total RNA was extracted from the tissues (50–100 mg) using Trizol reagent (TaKaRa Biotechnology Dalian Co., Ltd, China). Precipitated RNA was suspended in 20 μL RNase-free H_2_O, diluted to 2 ng/μL, and stored at -80 °C. RNA quality was assessed by formaldehyde denaturing gel electrophoresis. The concentrations and purity of RNA were determined spectrophotometrically (Nanodrop ND-1000, NanoDrop Technologies Co., Ltd, USA).

Total RNA was reverse transcribed into cDNA using a HiScript Q RT SuperMix for qPCR (+gDNA wiper) kit (Vazyme Biotech Co., Ltd, China), which includes a genomic DNA removal module. The reaction mixture (10 μL) for cDNA synthesis consisted of 2 μL 5qRT SuperMix II, 500 ng total RNA, and RNase-free H_2_O. The reaction was carried out at 25 °C for 10 min, 50 °C for 5 min, 85 °C for 5 min and the products were then stored at 4 °C.

### Real-time PCR primer design and quantitative fluorescence PCR

Using Primer Express 2.0, we designed *MYD88* primers based on the gene sequence deposited in GenBank. Primers were synthesized by Takara Biotechnology Dalian Co., Ltd. (China). *GAPDH* and *ACTB* were used as internal control genes to normalize the threshold cycle (Ct) values of other transcripts. The primer sequences used for amplifications of *MYD88, GAPDH*, and *ACTB* are listed in Table 1.

**Table 1:** Real-time PCR primer sequences.

Gene	Accession No.	Sequence (5′ → 3′)	Length
*MYD88*	NM_001099923.1	F: 5′-GCTGGAACAGACCAACTAT-3′	153
		R: 5′-TCCTTGCTTTGCAGGTAAT-3′	
*GAPDH*	NM_001206359.1	F:5′-ACATCATCCCTGCTTCTACCGG-3′	188
		R: 5′-CTCGGACGCCTGCTTCAC-3′	
*ACTB*	XM_003124280.3	F: 5′-TGGCGCCCAGCACGATGAAG-3′	149

Real-time PCR amplification was performed using a PCR kit (Vazyme Biotech Co., Ltd, China) in a 25-μL reaction mixture containing 2 μL of cDNA (500 ng), 0.5 μL of the forward and reverse primer (10 μM each), 0.5 μL of 50x ROX Reference Dye II, 10 μL of 2x SYBR Green Realtime PCR Master Mix, and ddH_2_O. PCR conditions were set at 95 °C for 5 min, followed by 40 cycles of 95 °C for 5 s and 60 °C for 34 s. Dissociation curve analysis was done after amplification. A peak melting temperature (Tm) of 85 ± 0.8 °C in the dissociation curve was used to determine the specificity of the amplification product. The Tm value for each sample was calculated from the average of triplicate technical samples. The 2^-ΔΔCt^ method was used to calculate relative gene expression (Oparina *et al.*, 2012).

### Statistical analysis

Statistical analyses were carried out using SPSS 17.0 software (SPSS Inc, USA). The multivariate general linear model (GLM) was used to determine differences in transcript levels among different developmental stages. Data are reported as means ± SD. Significance was set at *P* < 0.05.

## Results

### Purity and integrity of total RNA

We assessed RNA quality using denaturing gel electrophoresis and RNA quantity by measuring nucleic acid and protein concentrations. On the gel, we observed three bands, corresponding to 28S, 18S, and 5S. Based on gel electrophoresis results, there was no DNA contamination or significant degradation (data not shown). The A_260_/A_280_ ratios of the samples ranged from 1.9 to 2.0.

### Quantitative fluorescence PCR amplification and melting curves

The real-time PCR amplification and melting curves for *MYD88* were consistent between amplification reactions. A single specific peak for *MYD88* with no primer dimers or non-specific reaction products was obtained. The standard curves for *MYD88, ACTB*, and *GAPDH* revealed that the amplification efficiencies of the target and reference genes were almost the same; therefore, the 2^-ΔΔCt^ method was used (Figure S1).

### *MYD88* gene expression in different tissues and development stages

**Figure 1:**
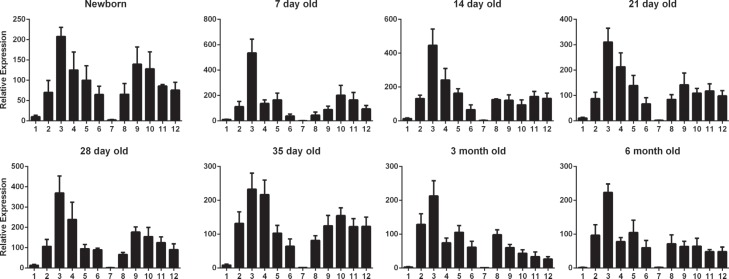
Expression profile of *MYD88* in tissues at different developmental stages, determined by the the 2^-ΔΔCt^ method. *MYD88* expression in the muscle of the 3-month-old group was set at 1.0. 1, heart; 2, liver; 3, spleen; 4, lung; 5, kidney; 6, stomach; 7, muscle; 8, thymus; 9, lymph nodes; 10, duodenum; 11, jejunum; 12, ileum.

Using the established SYBR green real-time quantitative PCR method, we measured the relative expression levels of *MYD88* in different tissues and at different development stages. *MYD88* expression levels were normalized to *ACTB* and *GAPDH* expression levels, and the expression of *MYD88* in the muscle of the 3-month old group was used as reference (calibrator) value (1.0). At all development stages, the average expression levels of *MYD88* in the spleen was the highest (Figure 1), while they were extremely low in heart and muscle. Expression was high in immunity-related tissues (liver, lung, kidney, thymus, and lymph nodes) and also digestive tract tissues (stomach, duodenum, jejunum, and ileum). Before weaning, *MYD88* expression was lower in the stomach than in the duodenum, jejunum or ileum.

### Analysis of *MYD88* expression at different development stages in the same tissue

**Figure 2:**
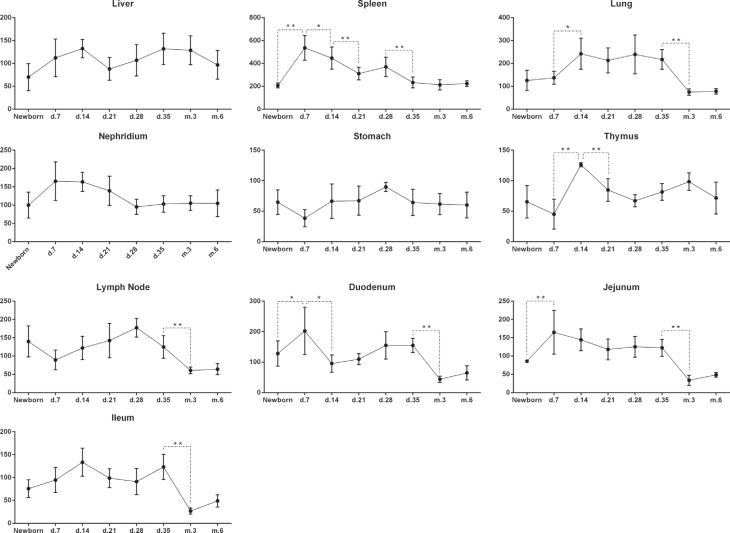
*MYD88* expression levels in each tissue of pigs at days (d.) 7, 14, 21, 28 and 35, and at months (m.) 3 and 5 *P < 0.05; **P < 0.01.

We analyzed *MYD88* expression in 10 tissues (excluding heart and muscle, which had extremely low *MYD88* expression levels during all development stages). Different tissues had different expression patterns during development (Figure 2). The expression of *MYD88* in the spleen, lung, and thymus was highest in 7-day old and 14-day old pigs and decreased in adulthood. Expression of this gene in lymph nodes was maintained at a high level, but declined from 28 days of age onwards. There was no significant difference in *MYD88* expression in the liver, kidney, and stomach during development. Expression of *MYD88* in intestinal tissues (duodenum, jejunum, and ileum) was high before 35 days (i.e., at weaning), with a peak from 7 to 14 days and decreasing post weaning. During adulthood the level was maintained low.

## Discussion

MYD88, an important transduction protein in myeloid cell differentiation, plays a vital role in the TLR signaling pathway. The TLR signaling pathway transfers signals via MYD88-dependent and MYD88-independent pathways. The MYD88-dependent pathway is the most important signal transduction pathway of TLRs (Serezani *et al.*, 2011). Studies have shown that MYD88 is indispensable for the downstream signal transduction of several TLRs. Specifically, the protein interacts with IRAK-4 (IL-1R-associated kinase-4), which subsequently activates other IRAK family members such as IRAK-1 and IRAK-2. This promotes the transfer of NF-κB into the nucleus and the activation of pro-inflammatory cytokine genes (Kawagoe *et al.*, 2008; Takeuchi and Akira, 2010). Kader *et al.* (2016) investigated the role of MYD88 using *MYD88* knockout mice and found that MYD88 plays a protective role in ehrlichiosis via the suppression of IL-10 and IL-17. Frantz *et al.* (2012) using *MYD88* knocked out mice enterocytes, found an increase in the number of intestinal mucus-associated bacteria and a decrease in the expression of polymeric immunoglobulin receptor (epithelial transport receptor of IgA) and mucin 2 (the main protein of intestinal mucus). In addition, the composition of the intestinal flora was significantly different between the enterocyte of *MYD88*-knockout mice and the wild-type mice, decreasing resistance to acute colitis in knockout mice. These results showed that the MYD88 signal might be essential for the homeostasis of the intestinal tract. Therefore, *MYD88* plays an important role in the immune response and defense system.

In this study, the analysis of the *MYD88* expression profile revealed that *MYD88* was expressed in all 12 tissues from Meishan pigs. The expression patterns changed over time, in agreement with Qiang (2008). *MYD88* expression was highest in spleen and higher in other immune tissues (liver, lung, kidney, thymus, and lymph nodes) and digestive tissues (stomach, duodenum, jejunum, and ileum) than in heart and muscle. *MYD88* is mainly expressed in T cells, B cells, NK cell lines, dendritic cell lines, thymus cell lines, and other immune cells, indicating that it may play an important role in immune responses (Hardiman *et al.*, 1996). Nishiya *et al.* (2007) observed that MYD88 is located in the cytoplasm and is not secreted. The authors hypothesized that the body wide expression of *MYD88* may be associated with the transduction of TLR signaling pathways. This may be the reason why *MYD88* showed a high expression level in most tissue samples from Meishan pigs, and hence we infer that the highest expression level of *MYD88* found in spleen may be related with the important function of spleen in innate and adaptive immunity.

Most studies on *MYD88* transcription were performed in humans and mice; few were done in livestock. Furthermore, there are no reports on the expression of *MYD88* in pigs in different developmental stages. This study analyzed the expression of *MYD88* in different tissues and at different developmental stages using real-time PCR. Piglets gain immune protection in two ways: passive immunization through sow milk and active immunization via their developing immune system (Salmon *et al.*, 2009; Levast *et al.*, 2014). Newborn piglets obtain specific antibodies from the sow colostrum, which can protect against infections (Ogawa *et al.*, 2016). However, maternal antibodies acquired from sow milk cannot be maintained over time (Levast *et al.*, 2014). After weaning, piglets experience psychological and physiological stress from being separated from the sow and leaving the familiar environment. The piglets may have problems adapting to the new environment, leading to conditional diarrhea.

The development of the digestive system is stimulated by early supplement feeding of 5 to 7-day old piglets (Wang *et al.*, 2002). In this study, the expression of *MYD88* increased in spleen, duodenum, and jejunum from birth to 7 days of age, which coincided with the feeding period. Expression of *MYD88* was significantly increased in the lung and thymus from 7 to 14 days of age. The small intestine (duodenum, jejunum, and ileum) has the highest absorptive ability (Caspary, 1992). Direct stimulation of the intestine may explain the increased expression in *MYD88* in the duodenum and jejunum after feeding. As the piglets aged, the expression level of *MYD88* in duodenum, jejunum and ileum showed a decreasing trend. Recent studies have shown that different parts of the intestine contain different microflora (Kelly *et al.*, 2017). Furthermore, the bacteria and function of the intestinal microflora may affect the level of inflammatory cytokines secreted by immune cells (Schirmer *et al.*, 2016). Therefore, the distribution of the microbial flora may affect the expression of *MYD88* and account for the discrepancies in *MYD88* expression patterns among the three intestinal sections.

Both the spleen and thymus are important immune organs. Expression of *MYD88* was significantly higher in the spleen than in the thymus, suggesting that the spleen may react more quickly in response to external stimuli compared to the thymus. The lung is a connective site between the organism and the environment and it is potentially susceptible to harmful substances and pathogenic bacteria. Therefore, *MYD88* expression significantly increased in the lung. The expression of *MYD88* was maintained at a relatively high level during early post-natal development, but decreased significantly in the heart, lung, lymph, duodenum, jejunum, and ileum from 35 days to 3 months of age and stabilized after sexual maturity. These results suggest that the high expression of *MYD88* during weaning probably enhanced the non-specific immune response and inflammatory response in piglets. As the immune system in Meishan pigs matures, the *MYD88* expression gradually decreases to a stable level.

This study explored the differences of *MYD88* expression in Meishan pigs at different development stages. We speculate that weaning stress and immune system development are the main reasons for the differences found. The high expression of *MYD88* may be beneficial for improving disease resistance in Meishan pigs. Future studies should examine the molecular mechanisms of *MYD88* regulation and develop breeding strategies for disease resistant Chinese pigs.

## Supplementary material

The following online material is available for this article:

Figure S1 - Amplification plot and melting curve analysis for *MYD88*.

## References

[B1] Arancibia SA, Beltrán CJ, Aguirre IM, Silva P, Peralta AL, Malinarich F, Hermoso MA (2007). Toll-like receptors are key participants in innate immune
responses. Biol Res.

[B2] Caspary WF (1992). Physiology and pathophysiology of intestinal
absorption. Am J Clin Nutr.

[B3] Frantz AL, Rogier EW, Weber CR, Shen L, Cohen DA, Fenton LA, Bruno ME C, Kaetzel CS (2012). Targeted deletion of MYD88 in intestinal epithelial cells results
in compromised antibacterial immunity associated with downregulation of
polymeric immunoglobulin receptor, mucin-2, and antibacterial
peptides. Mucosal Immunol.

[B4] Gay NJ, Symmons MF, Gangloff M, Bryant CE (2014). Assembly and localization of Toll-like receptor signalling
complexes. Nat Rev Immunol.

[B5] Hardiman G, Rock FL, Balasubramanian S, Kastelein RA, Bazan JF (1996). Molecular characterization and modular analysis of human
MYD88. Oncogene.

[B6] Janssens S, Beyaert R (2003). Functional diversity and regulation of different interleukin-1
receptor-associated kinase (IRAK) family members. Mol Cell.

[B7] Kader M, Alaoui-El-Azher M, Kode B, Kode B, McArthur M, Shinde A, Wells A, Ismail N (2016). MYD88 suppresses IL-10 and IL-17 production in response to
obligate intracellular Ehrlichia infection. J Immunol.

[B8] Kawagoe T, Sato S, Matsushita K, Kato H, Matsui K, Kumagai Y, Kawai T, Takeuchi O, Akira S (2008). Sequential control of Toll-like receptor-dependent responses by
IRAK1 and IRAK2. Nat Immunol.

[B9] Kelly J, Daly K, Moran AW, Ryan S, Bravo D, Shirazi-Beechey SP (2017). Composition and diversity of mucosa-associated microbiota along
the entire length of the pig gastrointestinal tract; dietary
influences. Environ Microbiol.

[B10] Levast B, Berri M, Wilson HL, Meurens F, Salmon H (2014). Development of gut immunoglobulin A production in piglet in
response to innate and environmental factors. Dev Comp Immunol.

[B11] Li X, Liu H, Shulin Y, Tang Z, Ma Y, Chu M, Li K (2009). Characterization analysis and polymorphism detection of the
porcine MYD88 gene. Genet Mol Biol.

[B12] Liu P, Qiu M, He L (2014). Expression and cellular distribution of TLR4, MYD88, and NF-κB in
diabetic renal tubulointerstitial fibrosis, in vitro and in
vivo. Diabetes Res Clin Pract.

[B13] Naro C, Sette C (2016). Dissecting a hub for immune response: Modeling the structure of
MYD88. Structure.

[B14] Nishiya T, Kajita E, Horinouchi T, Nishimoto A, Miwa S (2007). Distinct roles of TIR and non–TIR regions in the subcellular
localization and signaling properties of MYD88. FEBS Lett.

[B15] Ogawa S, Tsukahara T, Imaoka T, Nakanishi N, Ushida K, Inoue R (2016). The effect of colostrum ingestion during the first 24 hours of
life on early postnatal development of piglet immune systems. Anim Sci J.

[B16] Oparina NY, Sadritdinova AF, Snezhkina AV, Dmitriev AA, Krasnov GS, Senchenko VN, Melnikova NV, Belenikin MS, Lakunina VA, Veselovsky VA (2012). Increase in *NETO2* gene expression is a potential
molecular genetic marker in renal and lung cancers. Russ J Genet.

[B17] Parpaleix A, Amsellem V, Houssaini A, Abid S, Breau M, Marcos E, Sawaki D, Delcroix M, Quarck R, Maillard A (2016). Role of interleukin-1 receptor 1/MYD88 signalling in the
development and progression of pulmonary hypertension. Eur Respir J.

[B18] Qiang L (2008). Molecular cloning, tissues expression profile on porcine TLR7 and MYD88
and expression of MYD88 in pichia pastoris.

[B19] Salmon H, Berri M, Gerdts V, Meurens F (2009). Humoral and cellular factors of maternal immunity in
swine. Dev Comp Immunol.

[B20] Schirmer M, Smeekens SP, Vlamakis H, Jaeger M, Oosting M, Franzosa EA, Horst R, Jansen T, Jacobs L, Bonder MJ (2016). Linking the human gut microbiome to inflammatory cytokine
production capacity. Cell.

[B21] Serezani CH, Lewis C, Jancar S, Peters-Golden M (2011). Leukotriene B 4 amplifies NF-κB activation in mouse macrophages
by reducing SOCS1 inhibition of MYD88 expression. J Clin Invest.

[B22] Shchebliakov DV, Logunov DY, Tukhvatulin AI, Shmarov MM, Naroditsky BS, Ginzburg AL (2010). Toll-like receptors (TLRs): The role in tumor
progression. Acta Nat.

[B23] Singh MV, Cicha MZ, Chapleau MW, Abboud FM (2015). Interactions of MYD88 and TRIF-pathways of innate imune responses
regulate Angiotensin II hypertension. Circulation.

[B24] Su B, Luo T, Zhu J, Fu J, Zhao X, Chen L, Zhang H, Ren Y, Yu L, Yang X (2015). Interleukin–1β/Iinterleukin–1 receptor–associated kinase 1
inflammatory signaling contributes to persistent Gankyrin activation during
hepatocarcinogenesis. Hepatology.

[B25] Sun L, Xia RW, Yin XM, Yu LH, Zhu GQ, Wu SL, Bao WB (2015). Analysis of differential expression of TLR4 and TLR4 signaling
pathway genes under lipopolysaccharideinduced pig intestinal epithelial
cells. Chin J Anim Vet Sci.

[B26] Takeuchi O, Akira S (2010). Pattern recognition receptors and inflammation. Cell.

[B27] Tohno M, Shimazu T, Aso H, Kawai Y, Saito T, Kitazawa H (2007). Molecular cloning and functional characterization of porcine
MYD88 essential for TLR signaling. Cell Mol Immunol.

[B28] Wack A, Gallorini S (2008). Bacterial polysaccharides with zwitterionic charge motifs:
Toll-like receptor 2 agonists, T cell antigens, or both?. Immunopharmacol Immunotoxicol.

[B29] Wang WQ, Fan B, Wang LL, Cai TH, Huang K (2002). Effect of early supplementary feeding and ablactation on piglet
weight. Guizhou Agric Sci.

[B30] Zhou M, Duan Q, Li Y, Yang Y, Hardwidge PR, Zhu G (2015). Membrane cholesterol plays an important role in enteropathogen
adhesion and the activation of innate immunity via flagellin–TLR5
signaling. Arch Microbiol.

