# Supplementary material for: Spatiotemporal expression of MYD88 gene in pigs from birth to adulthood
Source: Genet Mol Biol. 2018 Jan 22;41(1):119–24. doi: 10.1590/1678-4685-GMB-2017-0014 (PMC5901504; doi:10.1590/1678-4685-GMB-2017-0014)
Supplement: Supplementary file 1 [file 1415-4757-gmb-1678-4685-GMB-2017-0014-Suppl01.pdf]

Supplementary Material to "Spatiotemporal expression of *MYD88* gene in pigs from birth to adulthood"

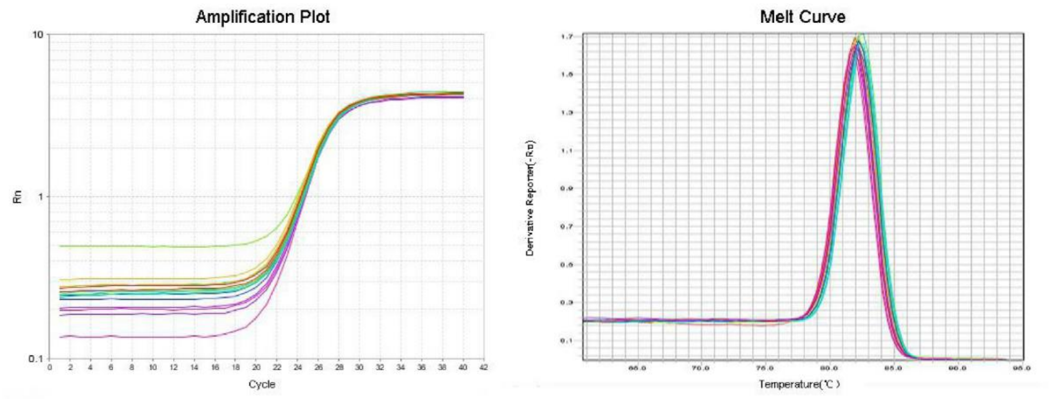

Figure S1 - Amplification plot and melting curve analysis for *MYD88*.
